# The Alveolar Gas Monitor: An Alternative to Pulse Oximetry for the Noninvasive Assessment of Impaired Gas Exchange in Patients at Risk of Respiratory Deterioration

**DOI:** 10.3390/jcm14165880

**Published:** 2025-08-20

**Authors:** W. Cameron McGuire, Eli Gruenberg, Tanner C. Long, Richa Sheth, Traci Marin, Brandon Nokes, Alex K. Pearce, Ann R. Elliott, Janelle M. Fine, John B. West, Daniel R. Crouch, G. Kim Prisk, Atul Malhotra

**Affiliations:** Health Division of Pulmonary, Critical Care, Sleep Medicine, and Physiology, University of California San Diego, La Jolla, CA 92093, USA; egruenberg5@gmail.com (E.G.); tclong@health.ucsd.edu (T.C.L.); richa.sheth@hsc.utah.edu (R.S.); tmarin@health.ucsd.edu (T.M.); bnokes@health.ucsd.edu (B.N.); apearce@health.ucsd.edu (A.K.P.); aelliott@health.ucsd.edu (A.R.E.); jfine@health.ucsd.edu (J.M.F.); jwest@health.ucsd.edu (J.B.W.); dcrouch@health.ucsd.edu (D.R.C.); kprisk@health.ucsd.edu (G.K.P.); amalhotra@health.ucsd.edu (A.M.)

**Keywords:** respiratory deterioration, respiratory failure, gas exchange, hypoxia, supplemental oxygen, alveolar gas monitor

## Abstract

**Background/Objectives:** The COVID-19 pandemic highlighted the limitations of pulse oximetry in detecting occult hypoxemia. The superiority of the alveolar gas monitor (AGM) compared to pulse oximetry (SpO_2_) in predicting respiratory deterioration among COVID-19-positive individuals has previously been demonstrated. Here, we combine COVID-19 and non-COVID-19 individuals as a combined cohort of participants to determine if the AGM has similar utility across a larger, more generalizable cohort. **Methods:** Adult patients (*n* = 75) at risk of respiratory deterioration in the emergency department (ED) underwent prospective assessments of their oxygen deficit (OD) and SpO_2_, simultaneously measured during quiet breathing on the AGM. The OD and SpO_2_ were then compared for their ability to predict the dichotomous outcome of the need for supplemental oxygen. The administration of supplemental oxygen was ordered by the clinical care team with no knowledge of the patients’ enrollment in this study. **Results:** In the logistic regression analysis, both SpO_2_ and OD significantly predicted the need for supplemental oxygen among COVID-19-negative individuals. However, in the multivariable regression, only OD (*p* < 0.001) significantly predicted the need for supplemental oxygen, while SpO_2_ (*p* = 0.05) did not in the combined cohort of COVID-19-negative and -positive individuals. Receiver operating characteristic (ROC) curve analysis demonstrated the superior discriminative ability of OD (area under ROC curve = 0.937) relative to SpO_2_ (area under ROC curve = 0.888) to predict the need for supplemental oxygen. **Conclusions:** The noninvasive AGM, which combines the measurement of exhaled partial pressures of gas with SpO_2_, outperforms SpO_2_ alone in predicting the need for supplemental oxygen among individuals in the ED at risk of respiratory deterioration regardless of the etiology for their symptoms (COVID-19-positive or -negative).

## 1. Introduction

The COVID-19 pandemic exposed unexpected limitations in the current healthcare system’s resource availability, both at the supply and personnel levels. The overwhelming burden that COVID-19 placed on the healthcare system underscored the need for efficient and safe tools to quickly triage and prioritize the care of patients at risk of respiratory failure. Although a very useful tool for assessing hypoxemia, pulse oximetry has many limitations compared to arterial blood gas (ABG) sampling, potentially resulting in inaccurate measurements due to metabolic disturbances, skin pigmentation, and vascular function [1].

We recently reported that the alveolar gas monitor, or AGM (MediPines, Yorba Linda, CA, USA), outperformed SpO_2_ as a noninvasive method for analyzing important physiological endpoints useful in predicting the risk of respiratory failure in patients with COVID-19 [2]. Such endpoints provide prognostic information that can be used to anticipate those who are likely to deteriorate versus those who are likely to recover spontaneously. The AGM is further advantageous because it does not require ABG sampling [3,4,5], making it highly accessible in under-resourced areas or in the field away from healthcare systems. However, the prior report stemmed from a study with a modest sample size, leaving a need for further data collection.

John B. West and others developed the AGM to obviate the need for ABG sampling in some cases as ABGs are invasive, costly, not without risk [6], and are being performed less frequently [7]. The AGM has been used with excellent accuracy and reliability in healthy outpatients, both young and old people, hypoxic individuals, volunteers exercising at altitude and in hypoxic conditions, patients with parenchymal lung disease in the outpatient setting, and COVID-19 patients with dyspnea in the ED. In these prior studies, the gPaO_2_ has correlated closely with the PaO_2_ from an ABG, and the OD has correlated closely with the AaDO_2_.

The AGM measures the exhaled tensions (partial pressure in mmHg) of oxygen (O_2_) and carbon dioxide (CO_2_), along with other parameters such as the respiratory quotient, oxygen saturation, respiratory rate, barometric pressure, inspired pressure of oxygen, and pulse rate. The O_2_ deficit (OD) is an AGM output that is used as a surrogate for the alveolar–arterial O_2_ gradient (AaDO_2_ or Aa gradient) based on the difference between the alveolar pressure of oxygen (PAO_2_) and the calculated arterial oxygen pressure (gPaO_2_). The calculation of gPaO_2_ makes use of measured pulse oximetry (SpO_2_), as detailed below in Section 2.

Use of the OD as a surrogate for the AaDO_2_ is relevant as ventilation–perfusion (VQ) disruption in the lung, specifically shunt and low VQ, will lead to widening of the Aa gradient due to lower PaO_2_ and increasing PAO_2_. By focusing on this difference, we are potentially more sensitive to VQ disruption than by being reliant on SpO_2_ alone. Moreover, even if SpO_2_ is adversely affected by the aforementioned factors, PAO_2_ is not; therefore, by focusing on a widened gradient, we are potentially more sensitive to disruptions in VQ. Abnormalities of the parenchyma or vasculature could lead to pathological VQ inequity, and this likely occurs prior to abnormalities on imaging. Therefore, in acute respiratory illnesses presenting with dyspnea, early sensitivity to VQ disruption could have diagnostic and therapeutic implications. Finally, the exhaled gas values may circumvent issues around skin pigmentation, a recent focus related to pulse oximetry limitations [1].

Despite the positive results in our AGM COVID-19 manuscript, with waning cases and ongoing debate as to whether COVID-19 respiratory failure is different from other causes of respiratory failure, we felt it was important to broaden our investigation to non-COVID patients. This is particularly germane as methods for efficient patient triage could be used in future disaster situations regardless of the etiology of respiratory failure.

Based on this conceptual framework, we sought to test the hypothesis that the OD was superior to SpO_2_ in predicting the need for supplemental oxygen in a real-world setting among patients with respiratory symptoms at risk of respiratory deterioration. Such findings would provide validation of our prior COVID-19 AGM report [2] and improve the generalizability of our findings to non-COVID-19 respiratory illnesses.

## 2. Methods

### 2.1. Study Design

This was a diagnostic accuracy study performed in a prospective fashion comparing the OD to SpO_2_ to predict respiratory decline, as defined by requiring supplemental O_2_ administration within 24 h after measuring the OD. Requiring supplemental oxygen was defined as any amount of oxygen support above room air, and included nasal cannulas, face masks, high-flow devices, noninvasive and invasive ventilation, and extracorporeal membrane oxygen. This study was approved by our Institutional Review Board, and all participants provided signed informed consent. The decision to administer supplemental O_2_ was made by the clinical care team, who had no knowledge of patients’ enrollment in this study. Further, the research team had no knowledge of the treating physician’s decision to administer supplemental O_2_. Administration of supplemental oxygen was ascertained by medical record review at least 24 h after measurement of the OD.

### 2.2. Participants

All patients were at least 18 years old and were approached for enrollment in the Emergency Department (ED) of two academic medical centers between November 2020 and November 2023. Patients were initially identified by screening the ED census for a chief symptom of dyspnea, as this is a symptom that is more likely to have impairments in gas exchange than, for example, abdominal pain. The medical record was then assessed for inclusion and exclusion criteria prior to approaching the patient for enrollment in this study. Inclusion criteria were afebrile adults who were not on chronic oxygen therapy with a recorded SpO_2_ (on the hospital pulse oximeter) < 97% on room air with a chief symptom of dyspnea. Exclusion criteria included being on any amount of supplemental oxygen, a resting SpO_2_ < 88% even if on room air, at-risk patients as defined by the Department of Health and Human Services Common Rule (i.e., 45 CFR 46 Subparts A–D), chronic O_2_ therapy, use of medications that could interfere with the accuracy of SpO_2_ (e.g., intravenous dyes, vasodilators, and vasopressors), facial trauma precluding the use of a nose clip, and advanced neuromuscular weakness preventing an adequate mouthpiece seal. It is important to note that the chief symptom of dyspnea or shortness of breath was only used to screen the ED patient board for possible candidates for this study, not to draw any conclusions about a patient’s dyspnea and their impairments in gas exchange. Of note, many patients had an increased O_2_ requirement between the time of medical record screening and the investigator’s arrival in the ED for enrollment, which precluded such patients from participating in this study. This issue, combined with investigator availability, necessitated convenience sampling. Participant safety was ensured by stopping the experimental protocol if a patient’s SpO_2_ fell below 88% on the hospital pulse oximeter, regardless of the SpO_2_ on the AGM.

### 2.3. Test Methods

In this diagnostic accuracy study, the index test (AGM) was compared to a reference test (pulse oximetry). The index variable of interest (OD) was compared to the reference variable of interest (SpO_2_), as displayed on the AGM.

### 2.4. Device Details

**Figure 1:**
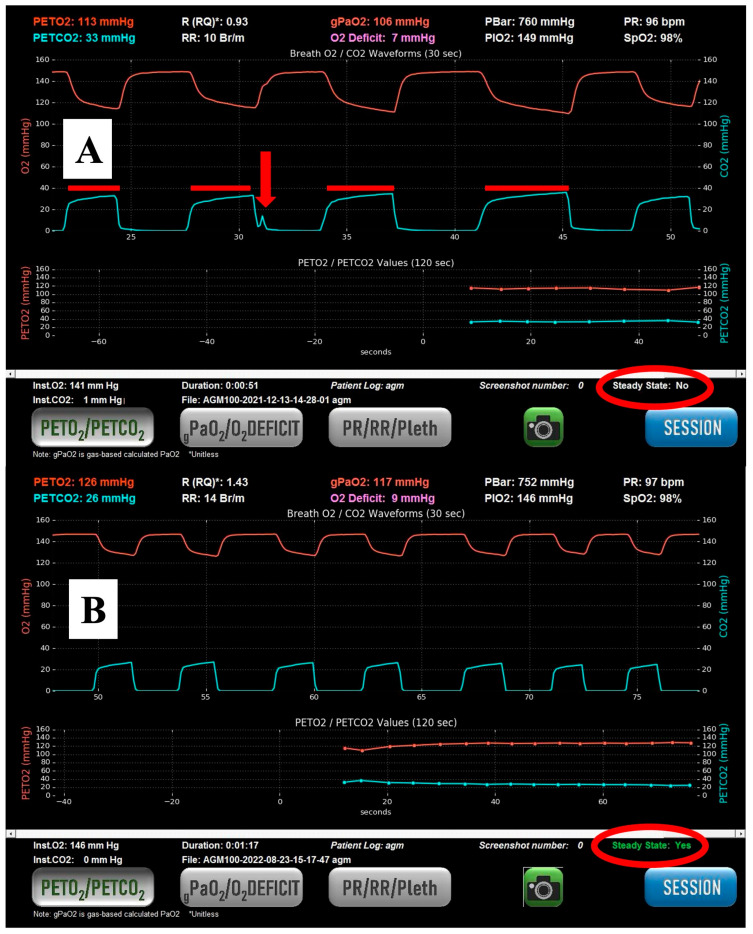
Example of breath-by-breath analysis as displayed on the AGM. (**A**) demonstrates a cough in the second breath (arrow) and variable expiratory times (red bars), resulting in a steady state not being achieved (red circle). (**B**) demonstrates similar breath-by-breath characteristics, resulting in the subject achieving a steady state (second red circle). The asterisk after R (RQ) stands for dimensionless number. It is a feature of the AGM100 that is part of the company (MediPines) programming of the device, not an asterisk to notify readers of additional information.

The expired pressures of CO_2_ and O_2_ are measured using rapid gas analyzers built into the housing of the AGM using a pump-driven, active sampling line connected at the midpoint of the mouthpiece, with data sampled at 100 Hz (see Figure 1). Infrared CO_2_ detection and fuel cell O_2_ analysis are used in the AGM to measure CO_2_ and O_2_ values, respectively.

### 2.5. Index Test Details

The AGM was used with the participant sitting semi-recumbent in bed to optimize functional residual capacity as much as possible, given their symptomatic state. Participants were instructed to perform quiet breathing as if they were reading a book to minimize breath-to-breath variability. If participants had difficulty maintaining a steady respiratory rate and depth, investigators played a metronome on their cellphones to aid participants in their respiratory pattern. Tidal breathing was performed on room air with a nose clip and a tight mouthpiece seal. The OD was derived by subtracting the calculated arterial partial pressure of oxygen (gPaO_2_) from the alveolar partial pressure of oxygen (PAO_2_). The gPaO_2_ was calculated from SpO_2_ rather than by direct measurement (PaO_2_), as is done with an ABG. Previous details about this calculation have been published by our group [8]. In summary, gPaO_2_ is an algebraic solution of the logarithm of the Hill equation using a Hill *n* of 2.88 for O_2_ based on work by Severinghaus [9,10,11]. The gPaO_2_ calculation also factors in the effects of PACO_2_ and, by proxy, PaCO_2_ on the oxygen tension at which point the O_2_–hemoglobin dissociation curve is 50% saturated (P50) based on research by Kelman [12,13,14]. The PAO_2_ was calculated by averaging end tidal O_2_ values from 5 consecutive breaths if and only if the patient and machine had reached a steady state [15] (see Figure 1).

Steady state was determined by the absence of variation in the end-tidal CO_2_ (etCO_2_) signal over the preceding 45 s. Stability in the etCO_2_ signal is contingent on the stability of the respiratory rate, tidal volume, and respiratory quotient. Prior research by our group has demonstrated good stability of etCO_2_ during steady-state breathing and is therefore usable in this context. A stable respiratory rate and depth (“steady state”) can usually be achieved in between one and four minutes. PACO_2_ was calculated much like PAO_2_, by averaging etCO_2_ values from five consecutive steady-state breaths. All the values from the AGM were saved in a .csv data file, exported onto a secure flash drive, and uploaded onto a research computer for analysis. This method obviated any transcription errors in recording values from the AGM.

A dummy continuous variable, ODFlip, was created by subtracting the OD from 100. Given that a higher OD is indicative of worse gas exchange, and a higher SpO_2_ is indicative of better gas exchange, ODFlip allowed plotting along the same axis in receiver operating characteristic (ROC) curves. Threshold values for ODFlip were then converted back to threshold values for the OD by subtracting ODFlip from 100.

### 2.6. Reference Test Details

The reference variable, SpO_2_, used for statistical analysis was obtained from the AGM rather than the SpO_2_ of the hospital pulse oximeter. This approach was chosen intentionally so that the only additional variables separating the OD from SpO_2_ were partial pressures of exhaled gases. For this reason, the superiority of the OD relative to SpO_2_ in predicting the outcome variable would support the clinical relevance of exhaled gas monitoring. SpO_2_ was also monitored using hospital pulse oximeters due to standard of care, and the decision to stop early was based on an SpO_2_ < 88% on the hospital pulse oximeter, irrespective of the SpO_2_ on the AGM. The hospital pulse oximeters used in this study were manufactured by three different companies (Massimo (Brisbane City, QLD, Australia), Nonin (Plymouth, MN, USA), and Welch Allyn (Auburn, NY, USA)).

### 2.7. Statistical Analysis

All statistical analyses were performed in R (Version 4.3.3) or SPSS (Version 29.0.2.0). Logistic regression analyses with Bonferroni correction were performed with a statistical significance threshold of *p* < 0.01. The informedness of the model was based on Youden’s J statistic in the ROC curve analysis.

## 3. Results

Among the non-COVID-19 cohort (*n* = 45), three patients desaturated to SpO_2_ < 88% after enrollment and during data acquisition on the AGM. Since they did not reach a steady state, a usable AGM data file was not generated, and they were censored from the analysis. The remaining non-COVID-19 patients (*n* = 42) were combined with our previously described COVID-19 patients (*n* = 30) into a combined cohort (*n* = 72) for all subsequent analyses. Several variables of interest [gender, age, self-reported race, self-reported ethnicity, and body mass index (BMI)] between those who did and did not have COVID-19 were investigated to assess the generalizability of this study (see Table 1).

Three binomial logistic regressions were performed to ascertain the effects of the continuous index variable (ODFlip) and the continuous reference variable (SpO_2_) on the likelihood that participants would require supplemental O_2_ (the dichotomous dependent variable). The first logistic regression was performed on COVID-19-positive participants (*n* = 30) from our original study [2]. The second was performed on COVID-19-negative participants (*n* = 42), and the third was performed on the pooled cohort of all participants (*n* = 72). The linearity of the continuous independent variables with respect to the logit of the dependent variable was assessed via the Box–Tidwell procedure [16]. Bonferroni correction was applied using all five terms in each model, resulting in statistical significance being accepted when *p* < 0.01. Based on this assessment, both independent variables were found to be linearly related to the logit of the dependent variable. In short, as SpO_2_ increases and ODFlip increases, the need for supplemental oxygen decreases. Conversely, as OD increases, the need for supplemental oxygen also increases (see Table 2).

In the COVID-19-negative cohort, for each one-point increase in OD, the odds of needing supplemental O_2_ increased by 1.14 (95% CI, 1.03 to 1.26) (see Table 3). In the overall cohort, for each one-point increase in OD, the odds of needing supplemental O_2_ increased by 1.17 (95% CI, 1.07 to 1.27) (see Table 4).

A multivariable logistic regression was then run in the combined cohort to evaluate ODFlip and SpO_2_ in combination, demonstrating strong statistical significance for ODFlip (*p* < 0.001) and borderline statistical significance for SpO_2_ (*p* = 0.05) in predicting the need for supplemental oxygen.

An ROC curve was generated to compare the overall discrimination of SpO_2_ and ODFlip on the need for supplemental O_2_ among COVID-19-negative participants (*n* = 42). The area under the ROC curve (AUROC) for ODFlip was 0.896 (95% CI, 0.712 to 0.966) with a Youden’s Index of 0.664 at an OD of 41 mmHg, resulting in a sensitivity of 0.840 and a 1–specificity of 0.176. The AUROC for SpO_2_ was 0.839 (95% CI, 0.800 to 0.993) with a Youden’s Index of 0.565 at an SpO_2_ of 94%, resulting in a sensitivity of 0.800 and a 1–specificity of 0.235.

An ROC curve was generated to compare the overall discrimination of SpO_2_ and ODFlip on the need for supplemental O_2_ among all participants (*n* = 72). The AUROC for ODFlip was 0.937 (95% CI, 0.885 to 0.990) with a Youden’s Index of 0.754 at an OD of 37 mmHg, resulting in a sensitivity of 0.811 and a 1–specificity of 0.057. The AUROC for SpO_2_ was 0.888 (95% CI, 0.812 to 0.964) with a Youden’s Index of 0.668 at an SpO_2_ of 94%, resulting in a sensitivity of 0.811 and a 1–specificity of 0.143.

**Figure 2:**
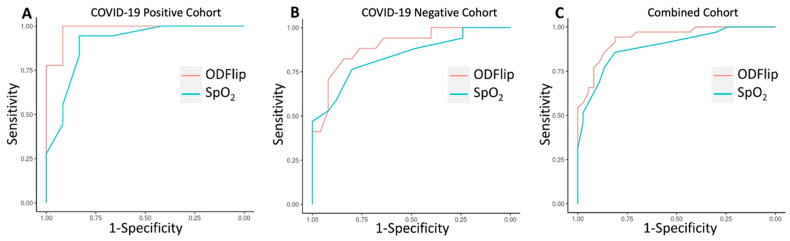
Receiver operating characteristic curves demonstrating the superior discriminative ability of an oxygen deficit to predict the need for supplemental oxygen as compared to pulse oximetry among COVID-19-positive participants (**A**), COVID-19-negative participants, and (**B**) all participants (**C**).

These two ROC curves demonstrate similar discrimination to the ROC curve previously published in COVID-19-positive participants [2] (see Figure 2). In all three ROC curves, the discriminative ability of the OD was statistically superior to SpO_2_.

## 4. Discussion

In this study, we have demonstrated that the OD can predict who will require supplemental oxygen based on univariable and multivariable regression and that it is superior to SpO_2_ for predicting this outcome based on the statistical parameters of receiver operating characteristic curve analysis.

This study is important for several reasons. First, we have extended our previous observations from COVID-19-positive patients by showing a significant AUROC for predicting respiratory deterioration via the AGM method in COVID-19-negative patients. In our cohort of non-COVID-19 patients, the etiologies of dyspnea included anemia, asthma, chronic obstructive pulmonary disease, granulomatous lung disease, heart failure, interstitial lung disease, pleural effusion, pneumonia (bacterial and non-COVID-19 viral), pulmonary embolism, and pulmonary hypertension among those patients whose etiology for their dyspnea could reliably be identified. This noninvasive tool may be a reasonable method to obviate the need for ABGs in some cases. For example, the AGM could be used in various clinical settings to help with care escalation and triage. In the outpatient clinic, it could be used to identify patients with mildly impaired gas exchange from their underlying chronic diseases. These baseline measurements could then be used as reference points for subsequent visits by these same patients. In the emergency department, it could be used to help with decisions about care escalation, such as providing supplemental oxygen, as we did in this study. On the hospital ward, it could be used for frequent, repeated measurements of gas exchange in hospitalized patients to assess for recovery from, or the worsening of, their acute respiratory illness. Moreover, in places without significant healthcare access (e.g., rural locations and under-developed countries), the AGM could be used in lieu of an ABG. Second, combining data from our pilot (COVID-19) dataset and confirmatory (non-COVID-19) dataset, our AUROC is outstanding and exceeds the highest threshold for discrimination according to Hosmer and Lemeshow [17]. We consider this finding an improvement upon the AUROC presented in our first report [2], where we demonstrated a trend toward significance but did not meet the threshold for statistical significance (*p* = 0.057) and had broad 95% confidence intervals (0.571 to 1.009) in the logistic regression. Moreover, the OD threshold with the maximal Youden’s index from our first manuscript is identical to the OD threshold in the combined dataset, with outstanding sensitivity, specificity, a positive predictive value, and a negative predictive value. Finally, the AGM has great potential to identify impending respiratory failure in a noninvasive manner, irrespective of underlying cause, which is of value in ongoing respiratory infectious diseases, as well as any future pandemics.

In prior studies, the gPaO_2_ has correlated closely with the PaO_2_, and the OD has correlated closely with the AaDO_2_ [2]. Use of the OD as a surrogate for the AaDO_2_ is relevant as ventilation-perfusion (VQ) disruption in the lung, specifically shunt and low VQ, will lead to widening of the Aa gradient due to a lower PaO_2_ and increasing PAO_2_. By focusing on this difference, we are potentially more sensitive to VQ disruption than being reliant on SpO_2_ alone. Finally, the exhaled values may circumvent issues around skin pigmentation, a recent focus related to pulse oximetry limitations, though more data are needed.

Various techniques have been used to triage patients at risk of respiratory failure. The Lung Injury Prediction Score (LIPS) was developed for acute lung injury (ALI) prediction in 2010 and 2011, prior to the Berlin definition of acute respiratory distress syndrome (ARDS) subsuming ALI in 2012. The AUROC in the single-center derivation study [18] for LIPS was 0.84, while the AUROC in the multi-center validation study [19] was 0.82. However, in the larger validation study, the sensitivity was 69% and the specificity was 78%. Finally, the model could only be applied to patients with a clearly defined risk factor for ALI/ARDS, thereby limiting its generalizability. More recently, the ratio of oxygen saturation (ROX) index has gained attention for its efforts to discriminate who would undergo intubation among patients requiring high-flow nasal oxygen for hypoxemic respiratory failure. Initially derived in 2016 [20], the ROX index had a modest predictive accuracy, with an AUROC of 0.74. The multi-center validation cohort of 2019 [21] demonstrated an AUROC of 0.759. However, the ROX index has limited applications for three reasons. First, it relies only on SpO_2_, which has the limitations described above. Second, it assumes the fraction of inspired oxygen based on the liters per minute of oxygen flowing through nasal cannula tubing. Third, it necessitates an already very ill patient needing a high degree of respiratory support before it can be applied for predictive modeling.

We and others have been using deep learning methods to identify patients at risk of respiratory failure. In Chest, Shashikumar et al. reported [22] excellent AUROCs (0.895 for derivation and 0.882 for validation) for all patients and even stronger AUROCs for COVID-19 patients (0.918 to 0.943 during prospective validation) for a deep learning model that consistently outperformed expert clinicians and established predictors, including the ROX index. The deep learning model was validated in COVID-19 and non-COVID-19 patients from two major academic medical centers. The implementation of this technique is ongoing but may well be supplemented with the use of the AGM to further improve the predictive models. Moreover, data enrichment using the AGM may improve actionability of the deep learning methods (e.g., clinicians could use the OD to determine optimal interventions prior to obvious respiratory deterioration).

Despite our study’s strengths, we acknowledge a few limitations. First, while noninvasive and rapid, the AGM requires patient engagement and some degree of stability in the respiratory rate and pattern. This issue limits its utility as a continuous, passive screening tool, such as the fingertip pulse oximeter; however, we believe it can still be used in a screening capacity by bedside nurses, respiratory therapists, and physicians at intervals, much like routine vital signs, and advocate for its addition to, not replacement of, pulse oximetry. Second, although our goal was to enroll a cohort with rich diversity, we ultimately included only ten black participants (13.7%) plus three of mixed race, which is more than predicted for San Diego County but could clearly be improved with multicenter studies. We further acknowledge that this is a single-center, two-site study, which may further limit its generalizability. Third, we did not perform an interventional study and thus cannot be confident which findings are clinically actionable. For example, future studies could attempt to identify patients likely to benefit from early empiric antibiotics or noninvasive ventilation prior to obvious clinical deterioration. Fourth, we chose “the need for supplemental oxygen” as a clinically important outcome but recognize that need for intubation or extracorporeal membrane oxygen (ECMO) may well have different predictors. Fifth, given the dynamic nature of patients at risk, we recognize that oxygen requirements within 24 h may differ for AGM readings taken early versus late in this window. However, we would argue that such random misclassifications should bias towards the null hypothesis, potentially making our statistically significant results somewhat stronger. Subsequent studies could assess multiple AGM readings over time, such that the trajectory of deterioration versus improvement could be more carefully defined. Despite these limitations, we view our findings as important, and they could potentially yield salient future studies.

## 5. Conclusions

The alveolar gas monitor, a noninvasive point-of-care device, was able to predict which patients with acute dyspnea in the ED would require supplemental oxygen with increased accuracy relative to pulse oximetry and regardless of their COVID-19 status. The addition of exhaled gas partial pressure to routine pulse oximetry (with an estimation of PaO_2_) adds value to the prognostic information provided by pulse oximetry alone and does so with a fidelity that exceeds or matches the accuracy of the best predictive tools for respiratory failure in the published literature.

## Figures and Tables

**Table 1:** Descriptive statistics of the COVID-19-negative and -positive cohorts.

Summary of COVID-19-Negative Cohort								Summary of COVID-19-Positive Cohort							
Variable	*n*	Mean	Std. Dev.	Min	Pctl. 25	Pctl. 75	Max	Variable	*n*	Mean	Std. Dev.	Min	Pctl. 25	Pctl. 75	Max
Participants	42							Participants	30						
Age (yrs)		60	16	19	51	72	84	Age (yrs)		61	14	34	52	72	85
BMI (kg/m^2^)		31	8.2	18	25	36	51	BMI (kg/m^2^)		29	6.7	20	24	32	49
Resp Rate		18	6.1	8	13	22	30	Resp Rate		23	17	7	13	27	99
Heart Rate		88	20	59	72	97	145	Heart Rate		81	11	60	73	89	100
Gender								Gender							
Female	13	31%						Female	12	40%					
Male	29	69%						Male	18	60%					
Race								Race							
Asian	1	2%						Asian	3	10%					
Black	6	14%						Black	4	13%					
Hispanic	5	12%						Hispanic	7	23%					
Other	3	7%						Other	0						
Native American	2	5%						Native American	0						
White	30	71%						White	23	77%					

**Table 2:** Univariable logistic regressions and Bonferroni corrections for all three cohorts, comparing OD, ODFlip, and SpO_2_ to the need for supplemental oxygen.

**COVID-19-Positive Cohort**						
**Est.**	**Std. Error**	**t-Value**	***p*-Value**	**Independent Variable (Predictor)**	**Model**	**Bonferroni Correction**
0.25	0.08	3.22	0.00	O2Deficit	SuppO2~O2Deficit	0.05
−0.25	0.08	−3.22	0.00	ODFlip	SuppO2~ODFlip	0.05
−0.74	0.26	−2.89	0.01	SpO_2_	SuppO2~SpO_2_	0.11
**COVID-19-Negative Cohort**						
**Est.**	**Std. Error**	**t-Value**	***p*-Value**	**Independent Variable (Predictor)**	**Model**	**Bonferroni Correction**
0.14	0.04	3.45	0.00	O2Deficit	SuppO2~O2Deficit	0.02
−0.14	0.04	−3.45	0.00	ODFlip	SuppO2~ODFlip	0.02
−0.58	0.19	−3.03	0.00	SpO_2_	SuppO2~SpO_2_	0.07
**Pooled Cohorts**						
**Est.**	**Std. Error**	**t-Value**	***p*-Value**	**Independent Variable (Predictor)**	**Model**	**Bonferroni Correction**
0.17	0.03	4.93	0.00	O2Deficit	SuppO2~O2Deficit	0.00
−0.17	0.03	−4.93	0.00	ODFlip	SuppO2~ODFlip	0.00
−0.67	0.15	−4.54	0.00	SpO_2_	SuppO2~SpO_2_	0.00

**Table 3:** Logistic regression results for COVID-19-negative participants demonstrating a statistical significance of an oxygen deficit in predicting the need for supplemental oxygen but in the absence of statistical significance for pulse oximetry.

	B	S.E.	Wald	df	Sig.	Exp(B)	95% CI	
							Lower	Upper
SpO_2_	−0.372	0.205	3.307	1	0.069	0.689	0.462	1.029
ODFlip	−0.132	0.053	6.276	1	0.012	0.876	0.791	0.972
Constant	42.415	19.507	4.728	1	0.030	2.63 × 10^18^		

**Table 4:** Logistic regression results for all participants demonstrating a statistical significance of an oxygen deficit in predicting the need for supplemental oxygen but in the absence of statistical significance for pulse oximetry.

	B	S.E.	Wald	df	Sig.	Exp(B)	95% CI	
							Lower	Upper
SpO_2_	−0.315	0.164	3.714	1	0.054	0.729	0.529	1.005
ODFlip	−0.153	0.045	11.299	1	<0.001	0.858	0.785	0.938
Constant	38.436	15.301	6.310	1	0.012	4.93 × 10^16^		

## Data Availability

The data that support the findings of this study are available from the corresponding author upon reasonable request.

## Abbreviations

The following abbreviations are used in this manuscript:

AaDO_2_	alveolar to arterial oxygen difference
ABG	arterial blood gas
AGM	alveolar gas monitor
AUC	area under the curve
AUROC	area under the receiver operating characteristic curve
CO_2_	carbon dioxide
COVID-19	Coronavirus Disease 2019
ED	emergency department
etCO_2_	end-tidal carbon dioxide
FiO_2_	fraction of inspired oxygen
gPaO_2_	calculated arterial partial pressure of oxygen
OD	oxygen deficit
O_2_	oxygen
PaCO_2_	partial pressure of arterial carbon dioxide
PACO_2_	partial pressure of alveolar carbon dioxide
PaO_2_	partial pressure of arterial oxygen
PAO_2_	partial pressure of alveolar oxygen
pH	acidity
SD	standard deviation
SpO_2_	oxygen saturation; pulse oximetry
ROC	receiver operating characteristic
VQ	ventilation–perfusion
